# Delirium Screening in Neurocritical Care and Stroke Unit Patients: A Pilot Study on the Influence of Neurological Deficits on CAM-ICU and ICDSC Outcome

**DOI:** 10.1007/s12028-020-00938-y

**Published:** 2020-03-20

**Authors:** Judith von Hofen-Hohloch, Carolin Awissus, Marie Michèle Fischer, Dominik Michalski, Jost-Julian Rumpf, Joseph Classen

**Affiliations:** Neurocritical Care and Stroke Unit, Department of Neurology, University Hospital Leipzig, Liebigstrasse 20, 04103 Leipzig, Germany

**Keywords:** Delirium, Neurocritical care, Stroke, CAM-ICU, ICDSC

## Abstract

**Background/Objective:**

Delirium is a common complication in critically ill patients with a negative impact on hospital length of stay, morbidity, and mortality. Little is known on how neurological deficits affect the outcome of commonly used delirium screening tools such as the Confusion Assessment Method for the Intensive Care Unit (CAM-ICU) and the Intensive Care Delirium Screening Checklist (ICDSC) in neurocritical care patients.

**Methods:**

Over a period of 1 month, all patients admitted to a neurocritical care and stroke unit at a single academic center were prospectively screened for delirium using both CAM-ICU and ICDSC. Tool-based delirium screening was compared with delirium evaluation by the treating clinical team. Additionally, ICD-10 delirium criteria were assessed.

**Results:**

One hundred twenty-three patients with a total of 644 daily screenings were included. Twenty-three patients (18.7%) were diagnosed with delirium according to the clinical evaluation. Delirium incidence amounted to 23.6% (CAM-ICU) and 26.8% (ICDSC). Sensitivity and specificity of both screening tools were 66.9% and 93.3% for CAM-ICU and 69.9% and 93.9% for ICDSC, respectively. Patients identified with delirium by either CAM-ICU or ICDSC presented a higher proportion of neurological deficits such as impaired consciousness, expressive aphasia, impaired language comprehension, and hemineglect. Subsequently, generalized estimating equations identified a significant association between impaired consciousness (as indexed by Richmond Agitation and Sedation Scale) and a positive delirium assessment with both CAM-ICU and ICDSC, while impaired language comprehension and hemineglect were only associated with a positive CAM-ICU result.

**Conclusions:**

A positive delirium screening with both CAM-ICU and ICDSC in neurocritical care and stroke unit patients was found to be significantly associated with the presence of neurological deficits. These findings underline the need for a more specific delirium screening tool in neurocritical care patients.

## Introduction

Delirium is an acute mental disturbance characterized by impairment of consciousness, attention, and perception, as well as changes in arousal, altered sleep–wake cycle, and presence of cognitive deficits such as memory dysfunction. It may present with psychotic features and emotional disturbance [1]. Delirium is a common complication in the Intensive Care Unit (ICU) setting and is associated with a longer hospital stay, higher morbidity and mortality, and a higher likelihood to be discharged to a nursing home [2, 3]. Regular delirium screening is, therefore, recommended by German and American guidelines in order to recognize and treat delirium early and prevent further complications [4, 5]. However, evaluation of delirium may be more difficult in patients with neurological disorders, since several characteristics of delirium resemble a broad range of neurological signs and symptoms [6, 7]. Studies investigating delirium in patients with stroke report an incidence rate of 10–48% [8]. Few data are available for delirium screening in patients with other (non-vascular) neurological conditions, such as in patients after epileptic seizures, or infections of the central nervous system [9].

The most frequently used instruments for standardized delirium screening on surgical and medical intensive care units are the Confusion Assessment Method for the Intensive Care Unit (CAM-ICU) [10] and the Intensive Care Delirium Screening Checklist (ICDSC) [11, 12]. In those settings, the CAM-ICU has a reported sensitivity of 75.5% to 80% and a specificity of 95.8% to 95.9%, while the ICDSC has a sensitivity of 74% to 80.1% and a specificity of 74.6% to 81.9% [13, 14]. However, both screening tools have been tested in neurological patients with less convincing results [15]: The CAM-ICU yielded sensitivity and specificity of 76% and 98%, respectively, in a cohort of stroke patients of which about 20% were excluded due to the level of consciousness after a large number of patients had been excluded for other reasons [16]. Furthermore, aphasia was identified as possibly leading to false positive results [16]. In one general intensive care cohort in the Netherlands, a specificity of only 17% in a mixed neurological/neurosurgical sub-cohort of 34 patients tested with CAM-ICU was reported [17]. In patients with mild to moderate traumatic brain injury, delirium was present in 45.9% of patients, but comparably low sensitivity and specificity for both tests were reported with 62% and 64% for CAM-ICU screening and 64% and 79% for ICDSC screening, respectively [18]. In 151 patients with ischemic or hemorrhagic stroke, subarachnoid hemorrhage, or cerebral tumors [19], delirium was diagnosed in 14% of patients according to ICDSC. In a more recent study from Denmark with a mixed neuro-intensive care cohort, sensitivity and specificity were 59% and 56% for CAM-ICU, and 85% and 75% for ICDSC, respectively [20]. Again, a significant number of patients had to be excluded or were not assessable due to a reduced level of consciousness [20].

Apart from aphasia, little is known about which neurological symptoms actually may compromise the utility of the screening instruments, though different neurological deficits such as aphasia, neglect, or dysphagia have been described as risk factors for developing delirium [21]. Here, we evaluated both CAM-ICU and ICDSC bedside delirium screening tools in a mixed stroke and neurocritical care patient cohort with special attention to existing neurological symptoms to guide future research in developing a delirium screening tool tailored to these patients.

## Methods

### Study Design

In this prospective, observational, single-center pilot study, we applied two of the most widely used instruments for delirium screening, the CAM-ICU and the ICDSC, in a mixed cohort of neurocritical care patients. The performance of the two screening instruments was assessed against delirium evaluation of the treating clinical team. Secondly, we also assessed patients according to the delirium criteria given by the International Classification of Disease N° 10 (ICD-10) [22]. Study reporting was in accordance with STROBE [23].

### Population and Setting

The study was conducted on the Neurocritical Care and Stroke Unit at the Department of Neurology at the University Hospital Leipzig. Approximately 1300 patients are admitted annually to the Neurocritical Care and Stroke Unit for treatment of neurovascular, epileptic, infectious, and neuromuscular conditions requiring stroke or intensive care treatment. The study was conducted over a period of 31 consecutive days (May 8 to June 7, 2017), representing a 1-month sample. As delirium incidence was unknown, sample size estimates were based on previous works that have studied delirium in stroke or neurocritical care [16, 18]. We estimated that a group size of some 100 patients with an expected delirium incidence of 20–26% [21] was required. To minimize bias, we aimed to include all patients consecutively. We estimated that data collection over a full month was required to complete the target cohort. Exclusion criteria were patients on palliative care and change of treatment to palliative care during the course of study. Patients who presented with a Richmond Agitation and Sedation Scale (RASS) ≤ − 4 were nevertheless screened daily for any change of consciousness, so this was not an a priori exclusion criterion. However, delirium screenings with a patient scoring a RASS of ≤ − 4 were later excluded in the analysis. Assessments were completed once daily within 24 h of admittance until discharge from ICU or death. Local ethics committee approval was obtained for this study (file number 242/17-ek). None of the routine practices (e.g., sedation, analgesia, or application of psychotropic medication) were modified during the course of the study.

### Delirium Evaluation

All senior physicians had certificates in neurology, which includes a 1-year training in psychiatry, as well as in neuro-intensive medicine, and had at least 5 years of experience working on the neurocritical care and stroke unit. The treating physicians (residents and senior physicians) were trained by receiving an interactive lecture on delirium symptoms and diagnostic criteria according to both ICD-10 and Diagnostic and Statistical Manual of Mental Disorders (DSM-V) delirium criteria. The clinical team was thus asked to evaluate the presence of delirium during afternoon rounds after having observed the patient for the duration of one shift and at approximately the same time on the weekends as there are no afternoon team rounds. Possible answers were: *Yes* (presence of delirium), *No* (absence of delirium), *Unsure*, and *Not Assessable* (according to the clinical team’s evaluation). The clinicians were blinded to the delirium screening with CAM-ICU and ICDSC.

### Screening Tools CAM-ICU and ICDSC

The CAM-ICU and the ICDSC were used to screen for delirium. The CAM-ICU consists of four consecutive items: For a positive delirium screening result, at least three items (either 1, 2, and 3 or 1, 2, and 4) must be positive. It evaluates the presence of delirium at the moment of screening. The ICDSC consists of eight items of which most are recorded by patient observation during a pre-specified time frame (8 to 24 h). A score of ≥ 4 points indicates delirium. We decided to use the ICDSC with a 24-h time frame. As part of the delirium screening, the level of consciousness was assessed by RASS, which ranges between a score of + 4 and − 5, where a score of 0 indicates a calm and alert patient, a score between + 1 and + 4 describes the level of agitation between restlessness and a combative condition, and a score between − 1 and − 5 indicates the level between drowsiness and coma. If RASS was ≤ − 4, the delirium screening was stopped for the respective day and reevaluated the following day.

### ICD-10 Delirium Research Criteria

We also recorded the ICD-10 research criteria at each patient visit [22]. If possible, all individual criteria were assessed according to bedside evaluation and previous chart documentation and otherwise rated as not assessable. (See Table 2 for further details.)

### Data Collection

Delirium screening with CAM-ICU, ICDSC as well as ICD-10 criteria was assessed daily for each patient on the ward by a resident (J.H-H., M.F., or C.A.) between 12 AM and 2 PM. Staff physician evaluation was done every day between 2 PM and 4 PM during afternoon rounds except for weekends. On weekends, the evaluation was done separately from rounds, but also shortly after the delirium screening was completed. The neurological deficits were recorded according to chart documentation. Information on the presence of infection, analgesia, sedation, and application of psychoactive medication as well as sociodemographic data including age, sex, past medical history with a focus on existing neurological disorders and previous cerebral insult current diagnosis, and length of hospital stay was collected on the basis of chart documentation.

### Bias

Clinical delirium evaluation was performed completely separately from the bedside screening tools by the clinical team which was blinded to the results of the bedside tests and the recorded ICD-10 research delirium criteria. Although screening with both CAM-ICU and ICDSC was completed one after the other by the same examiner, strict compliance with the evaluation protocol for CAM-ICU and ICDSC minimized the bias introduced by the same assessor’s evaluation. The ICD-10 delirium research criteria were recorded additionally as the last part of the examination by the same assessor because it was not feasible to assign a different independent evaluator to this task. However, to minimize bias, care was taken that the assessor was unaware of the results of the clinical team’s evaluation.

### Statistical Analysis

Descriptive statistics (frequency, percentage, and mean, where applicable) were used to describe the cohort and the delirium screening. Sensitivity and specificity, positive and negative predictive values, and positive likelihood ratios were calculated using a 2 × 2 table. Statistically significant differences with regard to the frequency of different neurological deficits and simultaneous presence of delirium were determined using generalized estimating equations (GEE) with bivariate variables to adjust for multiple measurements per patient. In the next step, GEE was used to predict the delirium screening outcome (for either CAM-ICU or ICDSC) by adjusting for various dependent variables such as the presence of dementia, sedative medication, and different neurological symptoms such as expressive aphasia, impaired language comprehension, and hemineglect. Additionally, we controlled for the presence of delirium by including the diagnostic reference (delirium evaluation by the clinical team) in the model as a dependent variable. Wald regression coefficients were determined with 95% confidence intervals and according to *p* values. *p* values < 0.05 were deemed significant. All calculations were done using SPSS 24 (IBM SPSS Statistic, IBM Corp.).

## Results

### Cohort Description

One hundred and twenty-three patients were admitted to the Neurocritical Care and Stroke Unit in the study period, and 644 screenings were conducted. The mean age was 69 years with a majority of male patients. Seventy-two (58.5%) patients were diagnosed with ischemic stroke, and five had an additional early epileptic seizure. Three patients (2.4%) were admitted with hemorrhagic stroke, 18 (14.6%) patients with transient ischemic attack (TIA), 11 (8.9%) patients with multiple epileptic seizures or status epilepticus, 4 (3.3%) with meningitis/encephalitis, and 15 (12.1%) with other diagnoses such as myasthenia gravis, migraine headache, paraneoplastic syndrome, or psychogenic seizures. For patient characteristics, see Table 1.

**Table 1:** Characteristics of 123 patients

Characteristics	Mean ± STD or number	Range or percentage
Age (in years)	68.9 ± 16.5	18–101
Sex	Male: 70	57%
	Female: 53	43%
*Previous neurological history*^†^		
Dementia	12	9.8%
> = 1 Ischemic stroke	23	18.7%
Intracranial hemorrhage	4	3.2%
Epilepsy	10	8.1%
Other	22	17.9%
None	101	82.1%
*Living situation*		
Home	111	90.2%
Nursing institution	12	9.8%
Nursing aid required	14	11.4%
*Diagnosis*		
Ischemic stroke	72	58.5%
Intracranial hemorrhage	3	2.4%
Transient ischemic attack	18	14.6%
Epileptic seizures/status epilepticus	11	8.9%
CNS infection	4	3.3%
Myasthenia gravis	3	2.4%
Migraine headache	3	2.4%
Others	9	7.3%
*Secondary neurological diagnosis*		
Early epileptic seizure after stroke	5	4.1%
None	118	95.9%
Length of stay (days)*	7.2 ± 9.7	1–66
Screening days/patient	5.3 ± 5.3	1–31
*Sedative medication/assessment*		
None	550	85.4%
During screening	81	12.6%
Given intermittently	13	2%
Patients ventilated	18	14.6%

*CNS* central nervous system, *STD* standard deviation

*Total length of stay on stroke/neurological intensive care unit may exceed average screening days as the study was conducted over a period of 31 days with patients being treated beyond the screening time frame

^†^Patients may have more than one previous neurological condition

### Delirium Incidence

The delirium incidence over the course of 31 days in the cohort was *n* = 23 (18.7%) according to the clinical team’s evaluation. The CAM-ICU delirium incidence amounted to *n* = 29 (23.6%), and the ICDSC delirium incidence was *n* = 33 (26.8%). There was no full overlap in delirium diagnosis between these two bedside screening instruments as in *n* = 10 patients, and delirium was diagnosed with one of the tools only. The delirium incidence was *n* = 16 (13%) according to ICD-10 criteria.

**Fig. 1:**
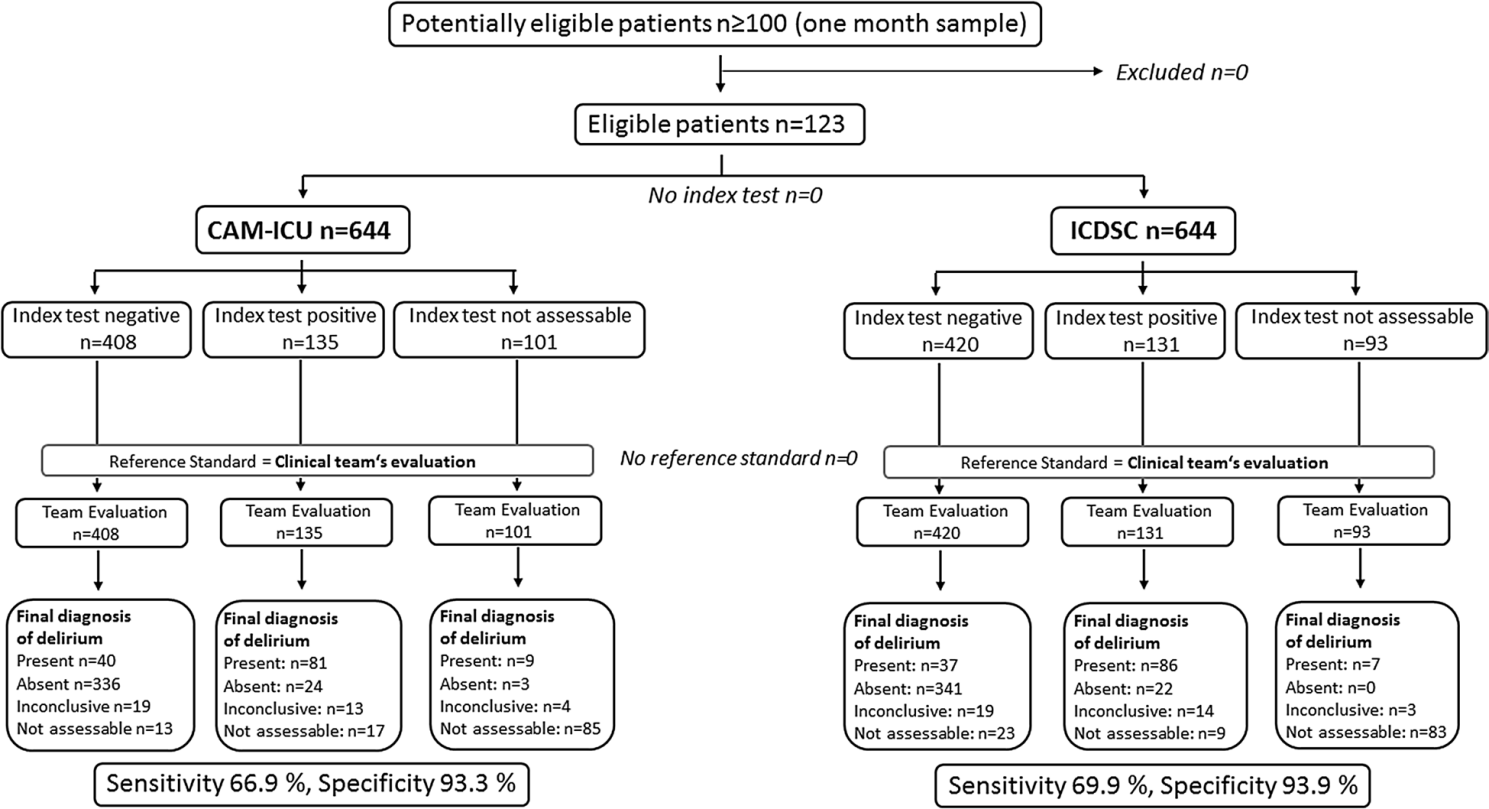
STARD flowchart [38]

Of 644 daily screenings, delirium was diagnosed in 130 assessments (20.2%) according to the clinical team’s evaluation, while 115 evaluations (17.9%) were rated as “not assessable” due to persistent coma and global aphasia. Using the test-based screening, a positive delirium evaluation was observed in 135 assessments (21%) with CAM-ICU and in 137 assessments (21.3%) with ICDSC. Evaluation with CAM-ICU and ICDSC was impossible in 101 (15.8%) and 93 (14.4%) of all screenings due to a RASS of ≤ − 4. The difference results from the fact that the CAM-ICU could not be rated in some patients due to global aphasia (Fig. 1).

In 107 screenings, patients presented with a RASS between < 0 and ≥ − 3, and in 93 screenings, patients were rated with a RASS ≤ − 4. Patients more often presented with a hypoactive (RASS < 0 and ≥ − 3, *n* = 47 (7.3%) assessments) than with a hyperactive delirium (RASS > 0, *n* = 15 (2.3%) assessments) during screening according to the clinical team’s evaluation.

All six ICD-10 criteria required in order to diagnose “delirium” were fulfilled in *n* = 48 (7.5%) evaluations, while 49 evaluations (7.6%) were rated as not assessable due to persistent coma. The most frequent ICD-10 criteria recorded were the underlying medical condition, followed by deficits in consciousness and attention, cognitive deficits and psychomotor abnormalities. In patients with a positive CAM-ICU or ICDSC delirium screening, between four and six ICD-10 delirium criteria were fulfilled. The most frequent variables which could not be assessed were “disturbance of cognition” (*n* = 147, 22.8%), followed by “disturbance of sleep or the sleep–wake cycle” (*n* = 145, 22.5%). (See Table 2 for further details.)

**Table 2:** ICD-10 delirium research criteria [1]—assessment outcome FO5 delirium, not induced by alcohol or other psychoactive substances

	Delirium criteria	Assessment positive	Assessment negative	Not assessable	Total
A	Clouding of consciousness, i.e., reduced clarity of awareness of the environment, with reduced ability to focus, sustain, or shift attention	*n* = 269 (41.8%)	*n* = 324 (50.3%)	*n* = 51 (7.9%)	*n* = 644 (100%)
B	Disturbance of cognition, manifest by both (1) impairment of immediate recall and recent memory, with relatively intact remote memory (2) disorientation in time, place, or person	*n* = 241 (37.4%)	*n* = 256 (39.8%)	*n* = 147 (22.8%)	*n* = 644 (100%)
C	At least one of the following psychomotor disturbances (1) rapid, unpredictable shifts from hypo-activity to hyperactivity (2) increased reaction time (3) increased or decreased flow of speech (4) enhanced startle reaction	*n* = 239 (37.1%)	*n* = 324 (50.3%)	*n* = 81 (12.6%)	*n* = 644 (100%)
D	Disturbance of sleep or the sleep–wake cycle, manifest by at least one of the following: (1) insomnia, which in severe cases may involve total sleep loss, with or without daytime drowsiness, or reversal of the sleep–wake cycle (2) nocturnal worsening of symptoms (3) disturbing dreams and nightmares which may continue as hallucinations or illusions after awakening	*n* = 417 (64.8%)	*n* = 82 (12.7%)	*n* = 145 (22.5%)	*n* = 644 (100%)
E	Rapid onset and fluctuations of the symptoms over the course of the day	*n* = 212 (32.9%)	*n* = 377 (58.5%)	*n* = 55 (8.5%)	*n* = 644 (100%)
F	Objective evidence from history, physical and neurological examination, or laboratory tests of an underlying cerebral or systemic disease (other than psychoactive substance-related) that can be presumed to be responsible for the clinical manifestations in A–D	*n* = 353 (54.8%)	*n* = 251 (39.0%)	*n* = 40 (6.2%)	*n* = 644 (100%)

*ICD-10* International Classification of Disease N° 10

Emotional disturbances such as depression, anxiety or fear, irritability, euphoria, apathy or wondering perplexity, disturbances of perception (illusions or hallucinations, often visual), and transient delusions are typical but are not specific indications for the diagnosis

### Sensitivity and Specificity

Sensitivity and specificity assessed against the clinical evaluation were 66.9% and 93.3% for screening with CAM-ICU and 69.3% and 93.9% for screening with ICDSC (Fig. 1). The positive predictive value was 0.77 for screening with CAM-ICU and 0.80 for screening with ICDSC, the negative predictive value was 0.89 for CAM-ICU and 0.90 for ICDSC, and the positive likelihood ratios (LR +) were 9.9 and 11.4, respectively.

### Confounding Neurological Variables

In patients who had a positive CAM-ICU or ICDSC screening result, neurological deficits such as expressive aphasia, impaired language comprehension, and hemineglect could be observed significantly more frequently in comparison with those patients who showed a negative screening result. Similarly, a positive delirium screening with CAM-ICU or ICDSC was more frequently associated with a RASS above or below 0. By adjusting for multiple assessments, these differences were in part significant for the individual symptoms. For a detailed description of neurological deficits, see Table 3.

**Table 3:** Neurological symptoms present during screening

Neurological deficits	Total of assessments *n* = 644	CAM-ICU				ICDSC			
		Positive *n* = 135	Negative *n* = 408	N/A^†^ *n* = 101	*p* value*	≥ 4 *p* *n* = 131	< 4 *p* *n* = 420	N/A^‡^ *n* = 93	*p* value*
Expressive aphasia	130 (20.2%)	42	52	36	0.033	38	62	30	0.016
Impaired language comprehension	147 (22.8%)	74	30	43	< 0.001	54	57	36	0.005
Hemineglect	91 (14.1%)	39	42	10	0.001	29	56	6	0.009
Hemiparesis	264 (41.0%)	69	149	46	0.017	67	156	41	0.053
Dysarthria	144 (22.4%)	41	99	4	0.845	45	96	3	0.124
Visual impairment	29 (4.5%)	10	18	1	0.942	9	19	1	0.417
*RASS*									
0	414 (64.3%)	38	374	–	–	37	376	–	–
≥ 1	30 (4.7%)	24	4	–	0.079	23	7	–	0.003
≤ − 1 ≥ − 3	107 (16.6%)	73	30	–	0.004	71	36	–	< 0.001
≤ − 4	93 (14.4%)	–	–	93	–	–	–	93	–

*CAM*-*ICU* Confusion Assessment Method in the Intensive Care Unit, *ICDSC* Intensive Care Delirium Screening Checklist, *RASS* Richmond Agitation and Sedation Scale: 0 = alert and calm, a score above 0 indicates restlessness up to agitated and aggressive behavior, a score below 0 indicates drowsiness up to light to moderate sedation, while a score of − 4 to − 5 stands for deep sedation to unarousable. Frequency *n* with (%)

*Adjusted for multiple assessments/patient by using generalized estimating equations

^†^*n* = 101 not assessable (N/A) due to a RASS of ≤ − 4 (*n* = 93) and severe aphasia (*n* = 8) at the time of testing

^‡^*n* = 93 not assessable due to a RASS of ≤ − 4

Next, generalized estimating equations were used to assess the influence of neurological deficits on delirium screening with CAM-ICU and ICDSC by adjusting for various variables that were observed significantly more often in patients with a positive CAM-ICU or ICDSC screening (Table 3). For each screening tool, a model with the clinical evaluation as diagnostic reference was established. The influential variables to predict a positive CAM-ICU result were “impaired consciousness” (as indexed by a RASS greater than 0 or between − 1 and − 3), “impaired comprehension,” and “hemineglect.” For a positive ICDSC, only the RASS showed a significant association (Table 4). Sedative medication and the presence of dementia were included in the model to control for other causes of changes in consciousness.

**Table 4:** Prediction of tool-based delirium screening

Parameter	CAM-ICU* (Regression coefficient, Wald 95% CI)	ICDSC^†^ (Regression coefficient, Wald 95% CI)
Dementia	− 1.32 (− 2.64–0.01); *p* = 0.051	0.34 (− 1.08–1.76), *p* = 0.64
*Impaired consciousness*		
RASS>0	3.60 (2.25–4.96), *p* < 0.001	4.15 (1.67–6.62), *p* = 0.001
RASS<0≥− 3	2.84 (1.52–4.15), *p* < 0.001	3.97 (2.54–5.40), *p* < 0.001
Expressive aphasia	0.92 (− 0.23–2.07), *p* = 0.12	0.97 (− 0.31–2.25), *p* = 0.14
Impaired comprehension	2.12 (1.26 − 2.98), *p* < 0.001	− 0.71 (− 2.06–0.65), *p* = 0.31
Hemineglect	1.51 (0.50–2.51), *p* = 0.003	− 0.38 (− 1.63–0.87, *p* = 0.55
Hemiparesis	0.01 (− 0.89–0.90), *p* = 0.99	− 0.12 (− 1.14–0.89), *p* = 0.81
Sedative medication	0.62 (− 0.69–1.93), *p* = 0.35	0.58 (− 2.00–3.17), *p* = 0.66

Model obtained with generalized estimating equations to assess for multiple measurements

*CAM*-*ICU* Confusion Assessment Method in the Intensive Care Unit, *ICDSC* Intensive Care Delirium Screening Checklist, *RASS* Richmond Agitation and Sedation Scale

*Assessments included in the model: *n* = 481 (*n* = 163 missing as rated “non-assessable” by either CAM-ICU or evaluation by clinical team), goodness of fit (corrected quasi-likelihood under the independence model criterion): 206.2

^†^Assessments included in the model: *n* = 486, (*n* = 158 missing as rated “non-assessable” by either ICDSC or evaluation by clinical team), goodness of fit: 226.9

## Discussion

In this prospective observational study, the delirium incidence of 18.7%, according to the diagnostic reference, was in accordance with the past reports with mostly neurovascular or trauma patients [8, 15, 21]. A similar delirium incidence in our more heterogeneous cohort may indicate that delirium occurs at largely similar frequencies in other neurological disorders with treatment on a neurocritical care unit. However, as the majority of patients in the present study suffered from neurovascular conditions, no firm estimates about the incidence in a non-stroke neurocritical care cohort can be made.

In our cohort, we found low sensitivity (66.9%) and acceptable specificity (93.3%) for screening with CAM-ICU. Values were below those reported in a selected cohort of stroke patients by Mitasova and co-workers [16], but above those reported in other studies with neurological/neurosurgical patients [15, 17, 18, 20]. The sensitivity (69.3%) and specificity (93.9%) of the ICDSC were comparable to those of CAM-ICU and similar to the findings in the study by Larsen and co-workers [20]. Application of the ICDSC was also found feasible in a previous Canadian multicenter study in patients with a variety of neurosurgical conditions [19]. In contrast, the ICDSC performed much worse in a study focusing on patients with mild to moderate traumatic brain injury [18].

We found that impaired consciousness (as indexed by RASS) was associated with a positive result of both screening tools. Although the potential effect of the level of arousal on assessing delirium in critically ill patients has been recognized before, the nature of the relationship between arousal and delirium remains incompletely defined [5]. We decided to include patients with a RASS of − 3 (20 assessments in our study) as validated for both screening tools [10, 11]. As delirium may present with a decrease in arousal [5] and as altered consciousness is regarded as a core feature of delirium, some authors recommend including altered arousal and not only changes in attention in this definition [24]. In the study by Mitasova and co-workers [16], a sub-cohort with impaired consciousness (RASS of − 0.30 ± 1.06) was evaluated in which sensitivity (85%) and specificity (97.1%) were clinically acceptable. However, patients with a reduced level of consciousness were excluded beforehand [16]. In contrast, the mean RASS score in our cohort ranged between − 0.75 (± 1.48) for positive CAM-ICU evaluations and − 0.85 (± 1.57) for ICDSC evaluations ≥ 4 points. This difference likely explains why both delirium screening tools did not perform as well in our study. This observation may also be supported by the fact that test specificity in delirium evaluation by CAM-ICU and ICDSC [18] tends to be lower in patients with impaired consciousness due to moderate traumatic brain injury and patients more deeply sedated. However, test sensitivity and specificity in the Danish study by Larsen and co-workers [20] which only evaluated patients with a minimal RASS of − 2 were even slightly worse for screening with CAM-ICU, though the test characteristics of ICDSC were similar to our study. Furthermore, patients unable to be assessed had a significantly lower Glasgow Coma Score and RASS than assessable patients [20]. Just as delirium, primary brain damage may primarily or secondarily lead to impaired consciousness. Neurocritical care patients with secondary deterioration of consciousness may, therefore, be more likely to receive a positive delirium screening label with either CAM-ICU or ICDSC. This is supported by the observation that sedation per se may result in a positive delirium screening [25], a notion, which, however, was not confirmed in our cohort. Additionally, sedation can lead to delirium itself [26, 27]. On the other hand, delirium may be overlooked in patients with primary brain damage as progressive impairment of consciousness may be misinterpreted as being part of the brain damage instead of an additional delirium. Moreover, drowsiness (defined as RASS of − 1) in non-neurologically ill patients is also associated with attention impairment and language abnormalities and increases the risk of delirium [28]. Therefore, patients with primary brain damage may be at even greater risk of developing delirium.

Patients with either a positive CAM-ICU result or ICDSC score of ≥ 4 points were more likely to present with severe focal neurological deficits such as expressive aphasia, impaired language comprehension, and hemineglect than patients with a negative result or a score of < 4. This is in line with other studies on delirium in stroke patients reporting more pronounced neurological deficits in patients with stroke and delirium [29, 30]. Neurological deficits such as hemineglect, dysphagia, or aphasia have previously been identified as risk factors for delirium in neurocritical care patients with stroke or subarachnoid hemorrhage [21]. However, to the best of our knowledge, the influence of individual neurological deficits on delirium screening itself has been studied in only two studies including selected stroke patients where aphasia was identified to impact the delirium screening result with either CAM-ICU [16] or ICDSC [31]. The latter, recently published study, raised the ICDSC cutoff to 5 instead of 4 points in order to improve test sensitivity and specificity [31]. When adjusting for various neurological symptoms and multiple measurements, impaired language comprehension and hemineglect appeared to have a significant association with CAM-ICU screening results. However, this was not true for the screening with ICDSC, where only impaired consciousness (as indexed by RASS) was significantly associated with screening results. Interestingly, expressive aphasia did not appear to significantly affect the screening outcome with either of the tools as observed in other studies [16, 31, 32]. However, screening with CAM-ICU was impossible in eight assessments due to global aphasia. In these cases, the ICDSC was still applicable due to its different construction as it is mostly based on observation, whereas the CAM-ICU is mostly based on interaction with the patient. Because neurological deficits (especially impaired comprehension) frequently impair the patient’s ability to interact with the environment, lesser dependence on interaction with the examiner may explain why ICDSC appeared to be more robust with regard to the presence of neurological deficits [32].

We consider it a strength of the study that all participating residents were trained in the recognition of delirium before the beginning of the study. As residents had bedside patient contact more frequently than senior staff physicians, prior training in delirium recognition may have counterbalanced the fact that senior physicians were more experienced. Another strength of this study is the fact that we consecutively screened all patients admitted to the neurocritical care and stroke unit over the time frame of 1 month, which may be representative of the mixed patient population in this setting and may be less biased in favor of certain specific conditions [16, 20, 30, 33]. Another strength may be the fact that we defined our reference as the result of a team-based evaluation, representing a pragmatic approach to evaluate delirium and discuss this decision whenever the evaluation was unclear or difficult.

We are also aware of certain limitations of the present study. Although the team was trained in the application of DSM-V and ICD-10 diagnostic delirium criteria before the initiation of the study, the individual delirium criteria leading to the decision of whether a delirium was diagnosed were not recorded by the team. Delirium screening could only be conducted once daily due to staff availability, although more reliable results may be obtained when screening is conducted multiple times daily [34]. By screening all patients within a single month, we have attempted to come close to an (ICU-)population-based investigation. As comatose patients could not be assessed, some residual selection bias may remain. Due to the single-center investigation, generalizability to other (neurocritical care) settings may be limited. Another limitation is the fact that both CAM-ICU and ICDSC were assessed consecutively by the same examiner every day. Because the screening methodology of CAM-ICU (based on interaction) and ICDSC (mostly based on observation) differ clearly, strict adherence to the study protocol has likely minimized this bias. An additional limitation is the pilot nature of this study, where study results are to be interpreted with caution [35].

In summary, our findings suggest that none of the existing popular screening tools detect delirium in neurocritical care patients with clinically acceptable sensitivity, although specificity was clinically acceptable. A clinically more useful delirium screening tool needs to account for the phenotypical overlap between neurological deficits and symptoms related to delirium. One possible strategy could be to combine observatory measures as in ICDSC with consideration of neurological deficits, which accounts for a patients’ inability to carry out certain tasks when (global or receptive) aphasia is present or accounts for inattention when hemineglect is present. As in neurological patients any signs of delirium could represent a signal for progressive brain injury, integrating results from technical investigations such as EEG, imaging, and laboratory testing may represent a promising approach to diagnose delirium secondary to acute brain injury [36]. Recently, signatures of delirium have been obtained by advanced analyses of EEG recordings in non-neurological patients [33, 37]. However, it remains unknown whether or not such measures are also applicable to neurological patients.

## Conclusions

The standard delirium screening tools validated in non-neurological settings are not reliable in patients with neurological deficits. Among neurological symptoms, impaired consciousness seems to have the greatest influence followed by language comprehension difficulties as well as hemineglect. The presence of neurological deficits should thus be specifically addressed in developing a bedside delirium screening tool that is tailored to the need of neurological patients.
